# Analysis of morbid obese diabetic and non-diabetic patients: clinical and social characteristics, habits and workup to undergo bariatric surgery in Lombardy, Italy

**DOI:** 10.1007/s00592-025-02604-1

**Published:** 2025-11-12

**Authors:** Antonio E. Pontiroli, Salvatore Guarino, Marta Tagliabue, Lucia La Sala, Lucia Centofanti, Stefano Olmi, Giuliano Sarro, Alessandro Giovanelli, Carmela Asteria, Elisa Galfrascoli, Umberto Mortola, Loredana Bucciarelli, Marco Antonio Zappa, Elena Tagliabue, Franco Folli

**Affiliations:** 1 Dipartimento di Scienze Della Salute, Università Degli Studi Di Milano, Milan, Italy; 2 IRCCS Multimedica, Milan, Italy; 3 Policlinico San Marco, Gruppo San Donato, Zingonia, Italy; 4 Università Vita-Salute San Raffaele, Milan, Italy; 5 Istituto San Gaudenzio, Novara, Italy; 6 IRCCS Galeazzi Hospital, Gruppo San Donato, Milan, Italy; 7 Department of Surgery, Fatebenefratelli Hospital, Milan, Italy; 8 Pio Albergo Trivulzio, Milan, Italy; 9 International Center for T1D, Pediatric Clinical Research Center Romeo Ed Enrica Invernizzi, Milan, Italy; 10 Diabetes and Metabolism Unit, ASST Santi Paolo E Carlo, Milan, Italy

**Keywords:** Bariatric surgery, Morbid obesity, Type 2 diabetes mellitus, Social characteristics, Education status

## Abstract

**Background:**

Studies on morbid obese diabetic and non-diabetic patients’ social characteristics and habits, in candidates for bariatric surgery (BS) are few. To gain further insights, we investigated 799 morbid obese diabetic (n = 111) and non-diabetic patients (n = 688).

**Methods:**

Family history of cardiometabolic diseases, personal history, education and occupation, comorbidities, daily habits, previous dietetic treatment, reasons and pathway to BS were investigated. Team members involved and examinations performed were also analyzed.

**Results:**

Family histories of obesity, diabetes and hypertension significantly associated with each other, and clinically overt diseases were also associated with family histories of the same disease, as diabetes and hypertension, and were more frequent in diabetic as compared to non-diabetic (p > 0.05, p < 0.0001 and p < 0.05). Females significantly differed from males for lower body mass index (BMI) (mean 41.2 vs 42.8 kg/m^2^), and a lower alcohol intake (p < 0.05 to p < 0.001). Knowledge about BS and reasons for BS varied according to age. BS was mostly requested for medical reasons (80.1%).

**Conclusions:**

Patients seeking bariatric surgery have a valid and well structured idea of obesity and are aware of the importance of eating less and physical activity in managing obesity, with differences linked to their educational levels. The interaction between physicians and surgeons improved the overall prognosis of patients seeking BS, based on screening of CV risk factors. Improved patients long term follow-up after surgery, identification of the suitable pre-BS diets, as well and identification of patients who might benefit from non-BS approaches, such as newer medical therapies could improve long term care of obesity.

## Highlights

Patients’ characteristics in candidates for bariatric surgery (BS) are not known, and we investigated in depth clinical and cultural background of 799 candidates.

Familial history of obesity, type 2 diabetes and arterial hypertension are significantly associated, and diseases (diabetes and hypertension) also associate with family history of the same diseases. Prevalence of cardiometabolic diseases significantly increased with age, and simultaneous presence of several diseases was observed.

Knowledge about BS and reasons for BS vary according to age, and BS is mostly requested for medical reasons.

Patients seeking bariatric surgery have a valid and well structured idea of obesity, with differences due to educational levels.

Identification of patients who might benefit either from BS or non-BS approaches, such as newer medical therapies, could improve long term care of obesity.

## Introduction

Obesity is the largest epidemics of non-communicable diseases, and severe obesity is extremely prevalent [1]. O besity significantly contributes to the development of cancer, cardiovascular diseases, liver diseases, and type 2 diabetes mellitus (T2DM) [2]. Bariatric surgery (BS) is the most efficient treatment of severe obesity, because of its superiority as compared with medical therapy (MT) [1], even though newer therapies based on GLP-1 agonists have reduced the gap between BS and MT [3–5]. In addition to its effect on weight, BS also produces improvements in T2DM with improved glycemic control and improvement or remission [6, 7], and can also prevents or delays incident T2DM [8]; BS is associated with substantially lower all-cause mortality rates and longer life expectancy than usual obesity management [9], significantly improves many cardiovascular parameters, as arterial hypertension [10, 11] and atrial fibrillation [12, 13] and obstructive sleep apneas (OSA) [14], but also alleviates symptoms of polycystic ovary syndrome [15] and improves mental health-related outcomes [16]. Access to BS should represent the final step of a protocol of treatment of obesity, based on collaboration among general practitioners, endocrinologists, internal medicine specialists, psychologists, psychiatrists, dietitians, nutritionists and surgeons [17]. However, there is also a biased approach to BS [18], and some general practitioners as well as specialty physicians are not aware of the possible benefits of BS and do not refer obese patients to surgeons [19, 20]. In addition, many patients prefer self-management of their own obesity, going from internet-based suggestions to opinions of friends, relatives, and patients who already underwent BS rather than referral to general physicians or to specialty physicians [21–23]. Negative perceptions regarding the safety and efficacy of BS are common in patients and referring practitioners, leading to overestimations of the risks or unrealistic expectations [24–26]. In Italy there are few data on the preferred pathways to undergo BS. In a previous study, performed for 6 months in three pilot regions (Lombardy, Lazio, Campania) charts of 2686 patients were evaluated by physicians and surgeons of the participating centers [27]. A chronic condition of obesity was evident for most patients, and most patients were self-presenting or referred by bariatric surgeons. Self-presenting patients were younger, more educated, more professional, and more mobile than patients referred by other physicians. Patients above the age of 40 years or with a duration of obesity greater than 10 years had a higher prevalence of diabetes, arterial hypertension and cardiovascular diseases [27]. The aim of this multicenter cross-sectional study, based on questionnaires and medical records, was to describe in detail: 1) the familial and educational characteristics of subjects undergoing BS; 2) information methods to know and to undergo BS; and 3) to describe in detail the diagnostic work-up of subjects undergoing BS.

## Methods

Five BS centers agreed to participate to the research protocol, and 799 subjects admitted to surgery units to undergo BS received a questionnaire during their hospital stay. Surgeons designated to practice BS and physicians taking care of the subjects received a different questionnaire. To protect the privacy of patients under study, data for each patient were categorized by blinded operators into classes, anonymized, and aggregated into databases, one for each center. Therefore, mostly categorical data were considered, with frequencies within each class, and a final aggregated database was analyzed for this study. The protocol of the QUECHI study was approved on February 21st 2019 by the Ethical committee of Area 1 Milano. The protocol was activated in 2020, but the COVID-19 pandemics caused several interruptions to the protocol (March and October 2020, March 2021, March 2022), and it was also necessary to amend the protocol (2020/M/233 of October 21st 2020), as administrative reasons forced some centers to quit the protocol, and new centers were involved. Enrollment period was completed at the end of 2022. All patients that underwent bariatric surgery, either primary or revisional surgery, were included; were excluded patients with lacking information about family history (about 100 patients). Informed consent was signed by all the participants.

### Statistical analysis 

Categorical variables were presented as frequencies and percentages and compared by Chi Square test or Fisher’s exact test, as appropriate. Continuous variables were summarized as mean and standard deviation and tested for normality distribution by Kolmogorov Smirnov test. Since normality was never met, continuous variables were compared by the non-parametric Wilcoxon test. Logistic regression models were implemented to evaluate association between subject characteristics and either path to, and reason for, and knowledge on BS. Analyses were performed with SAS Software v. 9.4. p-values < 0.05 were considered statistically significant for all analyses.

## Results 

 The overall details of patients considered for the study, i.e., sex, age and body mass index (BMI) distribution, education level, occupation, referring method, previous dietary treatments and previous bariatric surgeries, current habits (physical activity, diet, drug therapies), and associated diseases are reported in Table 1. Table 2 details the main differences between type 2 diabetic and non-diabetic patients. The vast majority of co-morbidities are significantly more frequent in diabetic subjects, as well as specialists’ visits and physical examinations.

**Table 1:** Baseline conditions of subjects in the study

Subjects (sex M/F)	799 (191/608)	Co-morbidities (%)		Treatment
Age (years)	43.5 ± 11.73	AH	218 (27.3%)	176 (22.0%)
BMI (kg/m^2^)	41.6 ± 6.69	Liver steatosis	112 (14.1%)	45 (5.6%)
Previous BS	54 (6.8%)	Hyperlipidemia	81 (10.1%)	49 (6.1%)
*Education*		OSAs	216 (27.0%)	145 (18.1%)
Elementary school	10 (1.3%)	Diabetes	111 (13.8%)	111 (13.9%)
Middle school	146 (18.3%)	Osteo-articular	121 (15.1%)	79 (9.9%)
High school and diploma	301 (37.7%)	Anxiety/Depression	129 (16.1%)	83 (10.4%)
Unknown	342 (42.8%)	CVD	72 (9.0%)	49 (6.1%)
*Occupation*		*The arrival to BS*		
No occupation/student	155 (19.4%)		*Knowledge on BS*	*Path to BS*
Employed	571 (71.5%)	From Friends	380 (47.6%)	327 (40.9%)
Unknown	73 (9.1%)	From Internet	100 (12.5%)	3 (3.8%)
Physical activity	236 (29.5%)	From Patients	422 (52.8%)	365 (45.7%)
Previous dietary attempts	713 (89.2%)	From GP	109 (13.6%)	132 (16.5%)
*Family history*		From Surgeon	46 (5.7%)	4 (5.0%)
Familial diabetes	449 (56.2%)	From Associations	30 (3.8%)	30 (3.8%)
Familial hypertension	428 (53.6%)	From Specialty doctors	–	46 (5.7%)
Familial obesity	511 (64.0%)			
*Reason for BS*				
Psychological	195 (24.4%)			
Medical	640 (80.1%)			
Social	283 (34.0%)			

Absolute frequencies (percentage) or mean ± SD

Percentages exceeding 100% are due to multiple choices items

*BS* Bariatric surgery, *BMI* Body mass index, *AH* Arterial hypertension, *OSAs* Obstructive Sleep Apnea Syndrome, *CVD* Cardiovascular disease, *Path* Way to arrive, *GP* General practitioner

**Table 2:** Differences between diabetic and non-diabetic patients

	Non-diabetic	Diabetic	p-value
Age (years)	42.75 ± 11.5	48.33 ± 10.93	< .0001
BMI (kg/m^2^)	41.68 ± 6.52	41.2 ± 5.07	0.9312
Intervention			0.0017
First	493 (90.46%)	95 (100%)	
Revision	52 (9.54%)	0 (0%)	
Physical activity	207 (34.33%)	25 (23.81%)	0.0341
Previous dietary attempts	285 (51.63%)	57 (64.77%)	0.0217
Family history			
Familial diabetes	344 (56.12%)	87 (81.31%)	< .0001
Familial hypertension	339 (56.88%)	72 (67.29%)	0.0442
Familial obesity	418 (68.30%)	78 (72.90%)	0.3429
Co-morbidities (%)			
AH	155 (25.45%)	53 (53.54%)	< .0001
OSAs	167 (27.33%)	48 (47.06%)	< .0001
Hyperlipidemia	48 (7.99%)	33 (33.33%)	< .0001
Liver steatosis	80 (13.2%)	31 (31.63%)	< .0001
CVD	46 (7.57%)	25 (26.04%)	< .0001
Osteo-articular	98 (16.09%)	20 (20.83%)	0.2474
Anxiety/Depression	109 (17.87%)	17 (17.35%)	0.9002
Reason for BS			
Psychological	179 (30.92%)	13 (12.5%)	0.0001
Medical	520 (89.81%)	100 (96.15%)	0.0395
Social	252 (43.52%)	29 (27.88%)	0.0028
Physician performing visits^a^			
Psychiatrist	248 (40.52%)	65 (60.75%)	< .0001
Endocrinologist	238 (38.95%)	64 (59.81%)	< .0001
Diabetologist	231 (37.75%)	71 (66.36%)	< .0001
Internist	253 (41.34%)	63 (58.88%)	0.0007
Dietologist^b^	317 (51.88%)	76 (71.03%)	0.0002
Neurologist (sleep specialist)	248 (41.2%)	68 (64.15%)	< .0001
Physical examinations^a^			
Chest X-ray	508 (83.28%)	101 (95.28%)	0.0014
ECG	456 (77.29%)	62 (59.05%)	< .0001
Polysomnography	281 (46.76%)	71 (67.62%)	< .0001
Spirometry	281 (48.36%)	28 (26.92%)	< .0001
GI X-ray	239 (39.24%)	64 (60.38%)	< .0001
Waist circumference	179 (30.92%)	8 (7.62%)	< .0001
Echocardiography	117 (20.28%)	39 (37.5%)	0.0001
Blood examinations^a^			
Lipases	332 (54.7%)	78 (72.9%)	0.0004
OGTT	237 (39.11%)	67 (62.62%)	< .0001

*BS* Bariatric surgery, *BMI* Body mass index, *AH* Arterial hypertension, *OSAs* Obstructive Sleep Apnea Syndrome, *CVD* Cardiovascular disease, *ECG* Electrocardiogram, *GI* gastrointestinal, *OGTT* Oral glucose tolerance test

^a^Only significant items are shown

^b^The dietologist is a physician with a post-graduate specialization in human nutrition and dietetics

### Subjects’ details

Family history was analyzed to evaluate simultaneous presence and type of other diseases. Familial obesity, diabetes mellitus and hypertension significantly associated each other. In addition, some diseases associated with family history of the same disease, as diabetes and hypertension (Table 3). Prevalence of diseases significantly increased with increasing age (Table 4). Simultaneous presence of several diseases was also observed. Prevalence of diabetes associates with arterial hypertension (AH), liver steatosis, cardiovascular disease (CVD), obstructive sleep apnea (OSA), hyperlipidemia, not with osteoarticular and depression. Also, AH associates with OSA, liver steatosis, hyperlipidemia, CVD, osteoarticular, but not with depression; finally, liver steatosis associated with hyperlipidemia, OSA, CVD, osteoarticular, not with depression (Table 5). Regarding employment status, the vast majority of male were employed while over 20% of female were unemployed. Furthermore, females had a lower alcohol intake, significantly lower BMI, lower frequency of hypertension and OSA, but a greater frequency of depression. In addition, women are less likely to receive treatment for AH and for OSA, and more likely to receive treatment for osteo-articular diseases and for depression (Table 6).

**Table 3:** Cross presence of family histories (FH) (obesity, type 2 diabetes—T2DM, arterial hypertension—AH) and frequency of diseases (T2DM, AH) per family histories

Familial history	Subjects	FH obesity	FH T2DM	FH AH	T2DM	AH
FH Obesity (+)	511	–	350 (68.5%)****	323 (63.2%)****	78 (15.3%)	148 (29.0%)
FH Obesity (−)	234	–	95 (40.6%)	103 (44.0%)	29 (12.4%)	68 (29.1%)
FH Diabetes ( +)	449	350 (78.0%)****	–	281 (62.6%)****	87 (19.4%)****	125 (53.4%)
FH Diabetes (−)	299	159 (53.2%)	–	146 (48.8%)	20 (6.7%)	93 (31.1%)
FH Hypertension (+)	428	323 (75.5%)****	281 (65.7%)****	–	72 (16.8%)*	175 (40.9%)****
FH Hypertension (−)	297	172 (57.9%)	148 (49.8%)	–	35 (11.8%)	38 (12.8%)

Significant differences are shown

( +): present; (−): absent

*p < 0.05; ****p < 0.0001

**Table 4:** Distribution of associated illnesses in different age quartiles. Absolute frequencies are shown

Age quartile	T2DM	AH	OSAs	CVD	HL	Os-Art	Dep	LS
1 (n = 173)	13	15	33	9	10	12	24	13
2 (n = 193)	19	39	53	14	16	18	35	26
3 (n = 191)	31	65	57	18	23	30	32	37
4 (n = 205)	44	94	69	29	31	57	32	35
*p-value*	0.0002	< .0001	0.0191	0.0095	0.0081	< .0001	0.8305	0.0068

Age quartiles: 1 = 1–33 years; 2 = 34–44 years; 3 = 45–52; 4 = 53+

*T2DM* Type 2 diabetes mellitus, *AH* Arterial hypertension, *OSAs* Obstructive sleep apnea syndrome, *CVD* Cardiovascular diseases, *HL* Hyperlipidemia, *Os-Art* Osteo-articular, *Dep* Depression, *LS* Liver steatosis

**Table 5:** Simultaneous presence of illnesses

1)							
Disease	AH (n = 218)	LS (n = 112)	OSAs (n = 216)	CVD (n = 72)	HL (n = 81)	Os-Art (n = 121)	Depression (n = 129)
T2DM (n = 107)	53	31	48	25	33	20	17
p-value	< 0.001	< 0.001	< 0.001	< 0.001	< 0.001	0.2474	0.9002

2)						
Disease	LS (n = 112)	CVD (n = 72)	OSAs (n = 216)	HL (n = 81)	Os-Art (n = 121)	Depression (n = 129)
AH (n = 218)	59	46	102	50	54	36
p-value	< 0.001	< 0.001	< 0.001	< 0.001	< 0.001	0.9814

3)					
Disease	CVD (n = 72)	OSAs (n = 216)	HL (n = 81)	Os-Art (n = 121)	Depression (n = 129)
LS (n = 112)	38	49	47	44	26
p-value	< 0.001	0.0002	< 0.001	< 0.001	0.0631

Absolute frequencies are shown and indicate subjects with presence of the two diseases

*T2DM* Type 2 diabetes mellitus, *AH* Arterial hypertension, *LS* Liver steatosis, *OSAs* Obstructive sleep apnea syndrome, *CVD* cardiovascular diseases, *HL* Hyperlipidemia, *Os-Art *Osteo-articular, *Dep* Depression

**Table 6:** Sex differences in subjects undergoing BS. Mean ± SD or absolute frequencies

	Men (n = 191)	Women (n = 608)	Significance (p)
Age (years)	42.4 ± 11.9	43.9 ± 11.7	0.1150
BMI (kg/m^2^)	42.8 ± 6.6	41.2 ± 6.2	0.0165
Profession			< .0001
No occupation	12 (7.02%)	132 (23.78%)	
Employee	158 (92.40%)	413 (74.41%)	
Student	1 (0.58%)	10 (1.80%)	
Wine	53 (36.3%)	106 (22.99%)	0.0002
Liquor	36 (23.68%)	55 (11.7%)	< .0001
AH	68 (39.08%)	150 (27.52%)	0.0039
OSAs	85 (48.3%)	131 (24.26%)	< .0001
Depression	12 (6.86%)	117 (21.63%)	< .0001
Treatment AH	56 (33.53%)	120 (23.26%)	0.0083
Treatment OSAs	55 (33.95%)	90 (17.89%)	< .0001
Treatment Os-Art	13 (8.18%)	66 (13.17%)	0.0908
Treatment depression	5 (3.11%)	78 (15.85%)	< .0001

*BMI* Body mass index, *AH* Arterial hypertension, *OSAs* Obstructive sleep apnea syndrome, *Os-Art* Osteo-articular

At univariate logistic analysis, age showed significant association with reasons for BS, with increased age less likely associated with psychological (OR = 0.98; p = 0.0286) reasons for BS (results not shown). Furthermore, highest grade of education showed significant positive association with psychological reasons for BS (OR = 5.68; p = 0.0373). At multivariable logistic analysis, independently of sex, psychological reasons were more common in more educated subjects (OR = 5.74; p = 0.0396) and in younger subjects (OR = 0.98 for one-year increase of age; p = 0.0238). Social reasons were more common in younger subjects, independently of sex and/or education, while medical and social reasons were not affected by ages and different education levels (results not shown). Information about BS deriving from friends was significantly more common among men than women (OR = 1.48; p = 0.0270). Similar result was obtained adjusting by age. Opposite trend was for information from operated subjects that was more likely among women, despite education and age. Knowledge deriving from internet use was more common among younger subsects, irrespective of sex, while knowledge deriving from GPs was more common for older subjects, irrespective of sex and of educational level (results not shown).

### Diagnostic work-up

For what regards specialists present in the team (Table 7), most specialists were contacted for most patients; other specialists were contacted for selected diseases (OSA, CVD, psychiatrist). This is reflected also by the instrumental work-up (spirometry, OGTT, etc.), where most exams were performed in most of patients while but some tests/visit were performed only in selected subjects (Table 8). Besides routine examinations performed (annotated) in almost all subjects, some blood examinations were annotated in selected subjects according to their presenting diagnosis. For instance, OGTT had been performed in 67 (62.6%) subjects with T2DM and in 237 (39.1%) subjects without T2DM (p < 0.001), and HbA1c had been analyzed in 46 (79.3%) subjects with T2DM, as opposed to 320 (69.7%) subjects without T2DM (p = 0.1300). Besides consultations performed in most patients, consultation with specialty physicians was performed (annotated) in selected subjects (Table 9). For instance, endocrinologists, internists, dietologists and diabetologists were involved in subjects with diabetes, CVD, OSA, hypertension and hyperlipidemia more than in patients without these diseases, and sleep specialists were involved in subjects with OSA, diabetes, CVD, AH and hyperlipidemia, more than in patients without these diseases; cardiologists were consulted in subjects with diabetes and OSA more than in patients without these diseases (results not shown). Polysomnography and echocardiography were requested more in subjects with than without OSA (p < 0.001), and echocardiography and Rx digestive were requested more in subjects with than without CVD (p < 0.001), and echocardiography was requested more in subjects with than without hypertension (p < 0.001) (data not shown).

**Table 7:** Description of the visits performed by the team

Physician performing visits	N = 799
Bariatric surgeon	494 (68.71%)
Psychologist	772 (96.98%)
Psychiatrist	338 (42.52%)
Dietitian^a^	664 (83.63%)
Endocrinologist	327 (41.18%)
Diabetologist	328 (41.26%)
Internist	341 (42.89%)
Dietologist^b^	420 (52.9%)
Pneumologist	176 (22.89%)
Cardiologist	307 (39.72%)
Neurologist (sleep specialist)	344 (43.88%)
Anesthesiologist	782 (98.61%)
Plastic surgeon	5 (0.63%)

^a^The dietitian has an undergraduate degree that includes courses comprising an approved didactic program in dietetics

^b^The dietologist is a physician with a post-graduate specialization in human nutrition and dietetics

**Table 8:** Examinations performed at entry, in order of frequency. On the left physical examinations, on the right blood examinations

Physical examinations	Number (%)	Blood examinations	Number (%)
Body weight	787 (99.37%)	Creatinine	788 (98.99%)
Body height	786 (99.37%)	AST	764 (96.34%)
BMI	785 (99.24%)	ALT	767 (96.60%)
Diastolic BP	785 (99.12%)	Total Cholesterol	710 (89.31%)
Heart rate	785 (99.12%)	HDL Cholesterol	708 (89.06%)
Systolic BP	782 (98.74%)	Triglycerides	703 (88.54%)
EGDS	775 (97.85%)	LDL Cholesterol	683 (86.02%)
Ultrasound abdomen	766 (96.72%)	Hba1c	415 (72.55%)
Chest X-ray	685 (86.49%)	Lipases	440 (55.7%)
ECG	577 (75.23%)	OGTT	329 (41.70%)
Polysomnography	418 (53.45%)	Amylases	144 (25.49%)
Spirometry	319 (42.14%)		
GI X-ray	330 (41.72%)		
Waist circumference	192 (25.40%)		
Echocardiography	166 (22.05%)		

*BP* blood pressure, *EGDS* esophagogastroduodenoscopy, *ECG* Electrocardiogram, *GI* gastrointestinal, *OGTT* Oral glucose tolerance test

**Table 9:** Frequency of specialist visits for patients with or without associated diseases

Associated disease (and specialist visit)	Presence of disease		Absence of disease		*p*
	Subjects	Visit	Subjects	Visit	
Diabetes and diabetologist	107	71 (66.36%)	612	231 (37.75%)	< .0001
Diabetes and endocrinologist	107	64 (59.81%)	612	238 (38.95%)	< .0001
Diabetes and internal medicine	107	63 (58.88%)	612	253 (41.34%)	0.0007
Diabetes and dietologist	107	76 (71.03%)	612	317 (51.88%)	0.0002
Diabetes and cardiologist	107	34 (32.69%)	612	219 (36.81%)	0.4205
Diabetes and psychiatrist	107	65 (60.75%)	612	248 (40.52%)	< .0001
Diabetes and neurologist (sleep specialist)	107	68 (64.15%)	612	248 (41.20%)	< .0001
CVD and diabetologist	71	48 (67.61%)	632	240 (37.97%)	< .0001
CVD and endocrinologist	71	47 (66.2%)	632	241 (38.19%)	< .0001
CVD and internal medicine	71	48 (67.61%)	632	254 (40.19%)	< .0001
CVD and dietologist	71	56 (78.87%)	632	325 (51.51%)	< .0001
CVD and psychiatrist	71	54 (76.06%)	632	246 (38.92%)	< .0001
CVD and neurologist (sleep specialist)	71	52 (73.24%)	632	250 (40.26%)	< .0001
OSAs and diabetologist	215	108 (50.23%)	497	186 (37.42%)	0.0014
OSAs and endocrinologist	215	109 (50.93%)	497	185 (37.22%)	0.0007
OSAs and internal medicine	215	110 (51.16%)	497	198 (39.84%)	0.0051
OSAs and dietologist	215	125 (58.41%)	497	261 (52.52%)	0.1477
OSAs and cardiologist	215	102 (48.57%)	497	150 (31.06%)	< .0001
OSAs and pneumologist	215	51 (24.29%)	497	116 (24.12%)	0.9619
OSAs and psychiatrist	215	109 (50.70%)	497	196 (39.44%)	0.0053
Hypertension and endocrinologist	217	96 (44.24%)	498	202 (40.64%)	0.3702
Hypertension and internal medicine	217	103 (47.47%)	498	209 (41.97%)	0.1729
Hypertension and dietologist	217	125 (57.60%)	498	264 (53.12%)	0.2683
Hypertension and pneumologist	217	61 (28.91%)	498	109 (22.61%)	0.0763
Hyperlipidemia and diabetologist	80	50 (62.50%)	618	235 (38.03%)	< .0001
Hyperlipidemia and endocrinologist	80	48 (60.00%)	618	237 (38.41%)	0.0002
Hyperlipidemia and internal medicine	80	44 (55.00%)	618	253 (40.94%)	0.0167
Hyperlipidemia and dietologist	80	63 (78.75%)	618	315 (51.05%)	< .0001
Hyperlipidemia and psychiatrist	80	49 (61.25%)	618	248 (40.13%)	0.0003
Hyperlipidemia and neurologist (sleep specialist)	80	48 (60.00%)	618	251 (41.35%)	0.0016

*CVD* cardiovascular diseases, *OSAs* obstructive sleep apnea syndrome

The vast majority of surgical interventions were laparoscopic (97.8%). The intervention was primary in 91.3% of subjects, and surgical techniques varied in primary and in revisional surgery. The most common intervention in primary surgery was laparoscopic sleeve gastrectomy (LSG) (88.8%), followed by roux-en-Y gastric bypass (RYGB) (6.5%); in revisional surgery RYGB was performed in 45.6%, followed by LSG in 35.1% and miscellaneous in 17.5%.

## Discussion

The main aim of this study was to analyze and report the familial and educational characteristics of subjects undergoing BS, their motivations and ways by which patients retrieve information about the surgical procedures and to undergo BS.

The association of familial histories with other familial histories is of interest, and these associations have never been described in morbid obesity, and there are no data on similar associations in non-obese subjects [28]. Also of interest is the association of individual familial histories with selected diseases, and this association has been described for several conditions [29].

For what regards patients’ clinical characteristics, we found that education was crucial in determining lifestyle of the subjects seeking BS. As a matter of fact, the most educated subjects showed better lifestyle, namely less smoking, more physical activity. Another important observation is that, in spite of a relatively young age of patients, several co-morbidities were present in both diabetic and non-diabetic patients, more frequent in diabetic than in non-diabetic patients.

As expected, age associated with diabetes, AH, OSA, hyperlipidemia, liver steatosis, CVD, osteoarticular diseases, but not with depression. Sex differences in lifestyle, diseases, and treatment were similar to what has been already described for the general population [30].

At multivariable analysis, psychological reasons were more common in younger subjects, while medical and social reasons were equally distributed among different ages and different cultural levels. In agreement with a previous study, informations acquired from GPs was more common for older subjects, irrespective of sex and of educational level, and informations deriving from the WEB was more common among younger subjects [27]. Of note, it was more common that more educated subjects would search informations from patients who had already undergone BS. The fact that not all patients underwent all visits is due to the different work-up, as several patients were screened before coming to BS centers. The diagnostic work-up was usually appropriate even in the presence of selected diseases; this aspect is of value, as shown under results, as patients with associated diseases underwent specialized visits more often than patients free of associated diseases.

## Strengths and limitations

Our results are in keeping with the knowledge that patients seeking bariatric surgery have a greater and more complex conceptualization of obesity than patients seeking behavioral/ pharmacological treatment and are aware of the importance of eating and physical activity in managing obesity, with differences linked to the educational level [27, 31]. Interaction between physicians and surgeons was important to improve the overall prognosis of patients seeking BS [32, 33], based on psychologic assessment [34], screening of CV risk factors [35], although the cost and the risk of attrition connected with multidisciplinary teams are greater than other approaches [36]. In the future, longer follow-up of patients, and identification of the suitable pre-BS diets [37], as well and identification of patients who might benefit from non-BS approaches, such as newer medical therapies are warranted [4, 5]. There are some limitations in this study. For instance the size of the study was relatively small, even because of the concomitant COVID-19 pandemic, and only 5 centers were involved, although these centers represent more than 60% of the BS procedures performed each year in Lombardy (average yearly number of BS procedures performed 2013–2018) [1]. Another aspect of this study is that the multidisciplinary approach is present in most centers, but with a preponderant role of surgeons. The preponderant role of surgeons probably contributes to the under-representation of other physicians. Family history data were collected by interviews, so recall bias cannot be excluded. Regarding between-center heterogeneity, there were some differences between centers probably due to the geographical localization of the different hospitals.

In conclusion, this study describes the real-world paths to BS for diabetic and non-diabetic morbid obese patients in Lombardy, Italy. Most of the patients are correctly included in the work-up for BS, with more than 50% patients self-report to BS, while patients referred by other physicians are only a minority. This can be due to an insufficient collaboration between surgeons and physicians and sould be substantially improved through mutual education, and a more collaborative interaction between GP and BS centers.

## Data Availability

Data are available from the corresponding authors upon reasonable request.
